# Supplementary material for: Genetic breakdown of a Tet-off conditional lethality system for insect population control
Source: Nat Commun. 2020 Jun 18;11:3095. doi: 10.1038/s41467-020-16807-3 (PMC7303202; doi:10.1038/s41467-020-16807-3)
Supplement: Supplementary file 1 — Supplementary Information [file 41467_2020_16807_MOESM1_ESM.pdf]

Supplementary Information

Genetic breakdown of a Tet-off conditional lethality system for insect population control

Zhao et al.

Supplementary Tables 1 and 2

Supplementary Figures 1 to 7

Supplementary Table 1. Effect of dietary tetracycline concentrations on DH-1 survival

Strain	Tet concentration (ug/ml)	No. Eggs	No. Pupae	No. Adults	% Eggs:Pupae	% Eggs:Adults
DH-1	0	200	0	0	0	0
	10	200	154	154	77%	77%
	50	200	162	161	81%	80.5%
	100	200	110	108	55%	54%
w[m]	0	200	152	151	76%	75.5%

Supplementary Table 2. Oligonucleotide PCR primer sequences in 5’ to 3’ orientation

AH1117	NGTCGASWGANAWGAA
AH1536	CGTTATAGTTCAAAATCAGTGACAC
AH1537	CTCGATATACAGACCGATAAAACAC
AH1540	GATCTGACAATGTTCAGTGCAG
AH1542	CTCTGGACGTCATCTTCACTTAC
AH1543	CTTTTATTATATACAGCCATAATGTCA
AH1554	GACACACGCGCAGACTGTC
AH1557	TATATGAGACGAGAGTAAGGGGTCC
AH1562	TTCGCCTTTTGTCGTTCTC
AH1565	TGAACCCGTGTATTTCGCAT
AH1566	AGTCTCTGCACTGAACATTGTCAG
AH1570	GCTTTACATTAGCCGCTCTT
AH1574	TATCGCACTCTGGACTCTGG
AH1577	AGTACTGTCATCTGATGTACCAG
AH1579	ACACTTACATACTAATAATAAATTCAACAAAC
AH1581	CATTGCAATTGGATCCATAT
AH1582	AGTACTTCTCGAGCTCTGTAC
AH1585	TATCTGAATTCTTCTTCCGTATT
AH1601	TCCAGGAACCCCAGTAGGTA
AH1610	GAATGGGCGGTACCTACTA
AH1614	GTCCAAACTCATCAATGTATCTTA
AH1615	TCCAAACTCATCAATGTATCAAG
AH1616	GAATTTCGAGTTTACCACTCCCTA
AH1619	ATGAACTCGACGCTACGTC
AH1631	ATCGAAATCGTCTAGCGCGT
AH1635	CTTCAAGATCCGCCACAA
AH1636	CATGTGATCGCGCTTCTC
AH1638	ACAACCACTACCTGAGCAC
AH1652	TGCTTGTCAATGCGGTAAGT

a) Driver integration site: *Tab2*: TAK1-associated binding protein 2 gene on chromosome 2R, locus 56C8/9 (FBgn0086358; Dmel_CG7417)

TATAACACCAAAATTACCCTTTATCTCGCAATATATTAATAAATACAAATCTCTAAAAGACGTC
ATAAATTAAAACGCGATTTAAATGACGTTTAAAGCGACTTCTAATTTATGTATGTTTATCACGA
ACTAAAAATTTAATTACTTTATAAATTTTAAAATTCTCAAGATAGTGAGCAATTATGTATGTTT
TAA<5’pBac_driver_3’pBac>TTAAAGAAAAAATTGTTGGAGTTCCACAAAATTTCGTTT
GAAAAATGCAGTTTTTATTTACCAAATATGCAAATCAAACGAAACTTAAAAGCTGACTTAGAAA
TCCTTTTGTTTATATGGGTGAAACTAAATAAATCCTTTTGTTTATGTAAATCACGTTCGATTGT
GTGCTTATTCCACGCATTAATAACTGCCTTACTCCATGCACAGAGTACAGCTTTTTTGAATGTC
ATGCTGATATGACAGAGCTGAAA

b) Effector integration site: *Sema2b*: Semaphorin 2b gene on chromosome 2R, locus 53C4 (FBgn0264273; Dmel_CG33960)

AAACGTCGCTTGGCCATTNGATGCGGACGTCGATGTGCACACGTTTAACCCACTGGCAAGTATT
CACACAAACAGAGCACTCGGAAAAATAAAAGATTGTGCAAGAGATAAGGAATCAAAAATGGGAA
ATATATATTATCATTTTGTAAACCTTTGCAATCGCAGACTGTCAATTTTAGTTCCAGGCTGAAA
TACCTTAAAAACTATACAATTCGAAAATGAACTTATTTAGAACATACAAAAACTGAAACTATTC
TATATTAATGTATCTCAATAAACAGCTTAAATGGCTTAAAAAAAGATACTCCTCAAAAAGAAAG
TACTTTTATTTCGAGTTGTAGAAAGTCCTGATAACTAACAAAAGAAGTTAATATGCTCATTAA<
5’pBac_effector_3’pBac>TTAAGTACTTTAAAGTATGTTAAGTATGTATCGCTTTAGCT
TATATTGCACTTTATGTGCTTATTTTCTAGCAGTGTACTTTAGTGAGCAGCACACACACTCTGT
GATAATTTTTCTGCTTTGTCGTGTGCCCTGGCTTTGCTCATGGCTCCAGTTCACAACGTCCTGG
GTCCTGGGCCCTGAGTCCTGAGTCCCGAGCTCCGAGTCCTGAGCCCCCTAAATGTTTATTGCTT
CATTCTGTTCATTCTGTTGCGCAATTTAATCGATGTCTGGCTGCTCGGCTGACTGCG

Supplementary Figure. 1. *tTA* driver and TRE-*hid*^{Ala5} lethal effector vector chromosomal insertion site sequences.
Line DH-1 **(a)** *tTA* driver (<*5’pBac_driver_3’pBac*>) and **(b)** lethal effector (<*5’pBac_effector_3’pBac*>) vector genomic chromosome 2R integration site sequences. TTAA sequences (bold blue) are the canonical duplicated insertion site for the *piggyBac* vector; maroon sequences indicate flanking genomic sequences identified by a FlyBase Blastn alignment to the *D. melanogaster* genome assembly, and black sequences immediately flanking the vector transgene insertions are of unknown origin, but exist in the non-transgenic *w*[m] host strain (and another *white* strain, w^{1118}), and were not introduced with the transgene vectors.

a

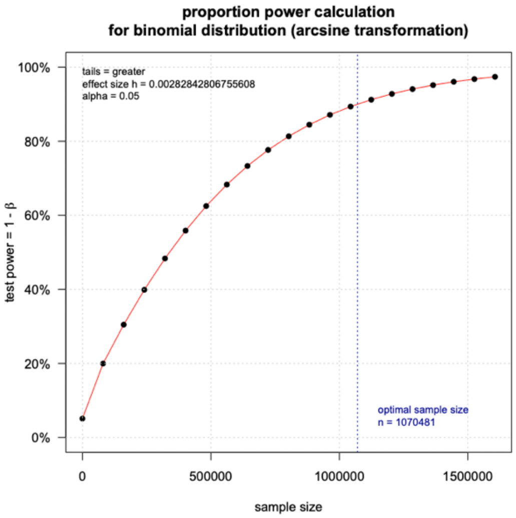

b

```
> pwr.p.test(h = ES.h(p1 = 0.000002, p2 = 0.0),
+         sig.level = 0.05,
+         power = 0.9,
+         alternative = "greater")

     proportion power calculation for binomial distribution
(arcsine transformation)

          h = 0.002828428
          n = 1070481
  sig.level = 0.05
      power = 0.9
alternative = greater
```

c

```
> pwr.p.test(h = ES.h(p1 = 0.0000058, p2 = 0.000002),
+         sig.level = 0.05,
+         n = 1200000,
+         alternative = "greater")

     proportion power calculation for binomial distribution
(arcsine transformation)

          h = 0.001988214
          n = 1200000
  sig.level = 0.05
      power = 0.7030269
alternative = greater
```

d

```
> pwr.p.test(h = ES.h(p1 = 0.00002, p2 = 0.0000058),
+         sig.level = 0.05,
+         n = 1200000,
+         alternative = "greater")

     proportion power calculation for binomial distribution
(arcsine transformation)

          h = 0.004127659
          n = 1200000
  sig.level = 0.05
      power = 0.9979912
alternative = greater
```

Supplementary Figure. 2. Power calculation to test for required sample size.
A power calculation using a one-sided Power P-test (pwr.p.test) was conducted to test for the required sample size for detecting primary-site genetic alterations based on the typical null mutation rate of 10^{-6} observed in *Drosophila*. If a power of 0.9 at the 0.05 significance level is required, then at least 1,070,481 double-heterozygous F_1 progeny should be screened for detection of primary-site mutations at either each or both loci for the binary lethality system **(a, b)**. Thus, the actual screened sample size of n=1,200,000 double-heterozygous F_1 progeny, estimated from control experiments, having a power of 0.93 resulted in a high significance level. The estimated frequency of 5.8 x 10^{-6} for primary-site mutations falls above the null mutation frequency of 10^{-6} and below a 10^{-5} frequency with a power of 0.7 **(c)** and 1.0 **(d)**, respectively, at the 0.05 significance level given the screened population size.

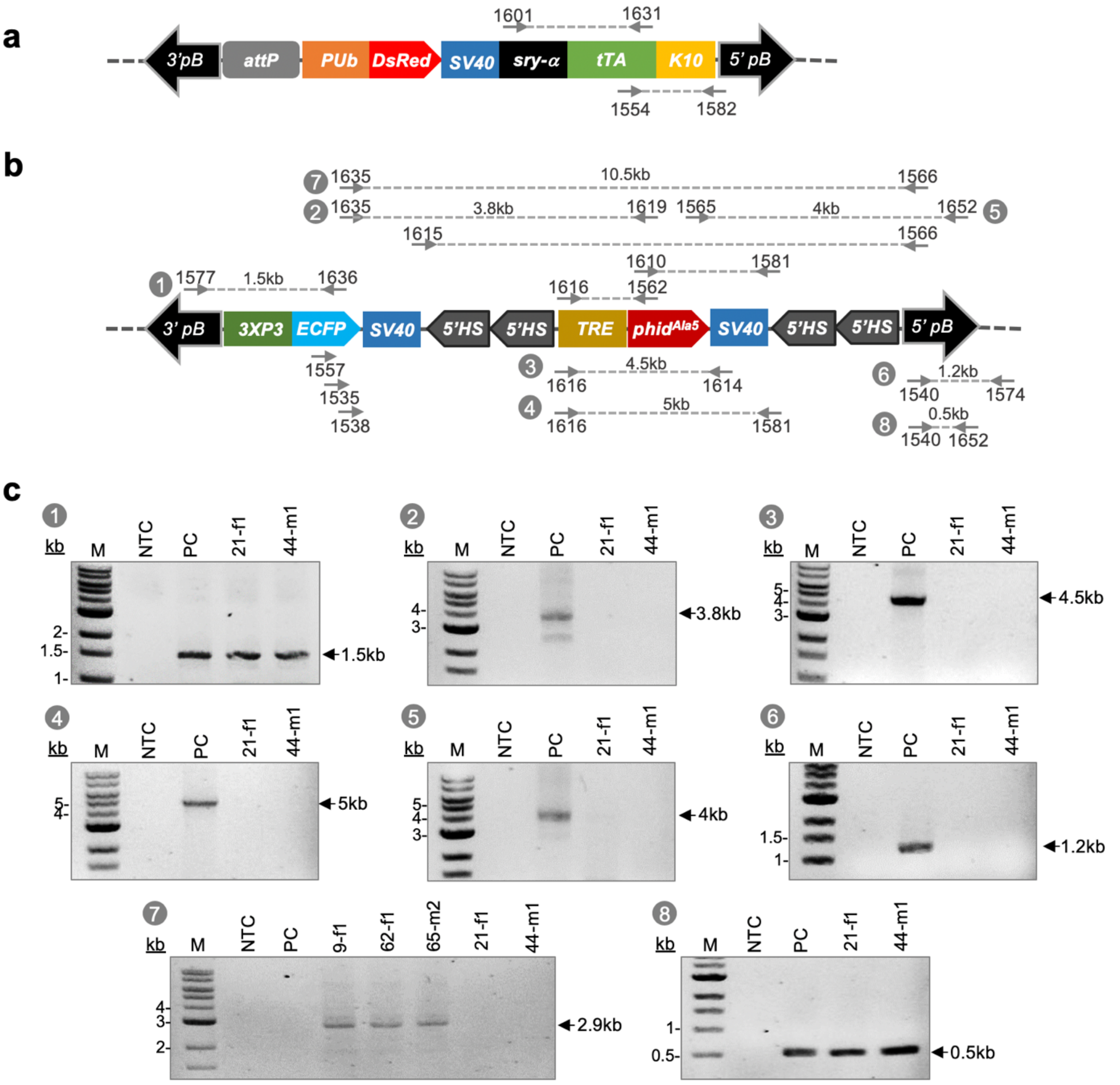

Supplementary Figure. 3. PCR analysis for mutations in the primary-site *tTA* driver and lethal effector cassette sequences.

Schematic map (not to scale) of the *tTA* driver **(a)** and lethal effector **(b)** vectors with PCR primers and numbered primer sets and their relative positions, and **(c)** corresponding PCR primer set products for the indicated lethal revertant lines. For primer set #7, 2.9 kb PCR products were only recovered in the internally deleted lines and not in the DH-1 control template (PC) due, presumably, to the 10.5 kb expected length and repetitive *5′ HS4* sequences. kb, DNA ladder; M, 1 kb markers; NTC, no template control; PC, DH-1 genomic DNA positive control.

a

```
tTA       TTGTATTGGCATGTAAAAAATAAGCGGGCTTTGCTCGACGCCTTAGCCATTGAGATGTTA    180
18f1      TTGTATTGGCATGTAAAAAATAAGCGGGCTTTGCTCGACGCCTTAGCCATTGAGATGTTA    420
          ************************************************************

tTA       GATAGGCACCATACTCACTTTTGCCCTTTAGAAGGGGAAAGCTGGCAAGATTTTTTACGT    240
18f1      GATAGGCACCATACTCACTTTTGCCCTTTAGAAGGGCAAA--------------------    460
          ************************************ ***

tTA       AATAACGCTAAAAGTTTTAGATGTGCTTTACTAAGTCATCGCGATGGAGCAAAAGTACAT    300
18f1      -------AAAAAAGTTTTAGATGTGCTTTACTAAGTCATCGCGATGGAGCAAAAGTACAT    513
                   ***************************************************
```

b

```
Hid        AGGACAAAAAGGAAGCCAGCACACACACACACACCCACACAATGGCCGTGCCCTTTTATT    420
45f1       AGGACAAAAAGGAAGCCAGCACACACACACACACCCACACAATGGCCGTGCCCTTTTATT    420
           ************************************************************

Hid        TGCCCGAGGGCGGCGCCGATGACGTAGCGTCGAGTTCATCGGGAGCCTCGGGCAACTCCT    480
45f1       TGCCCGA--------------------------GTTCATCGGGAGCCTCGGGCAACTCCT    454
           *******                          ***************************

Hid        CCCCCCACAACCACCCACTTCCCTCGAGCGCATCCTCGTCCGTCTCCTCCTCGGGCGTGT    540
45f1       CCCCCCACAACCACCCACTTCCCTCGAGCGCATCCTCGTCCGTCTCCTCCTCGGGCGTGT    514
           ************************************************************
```

Supplementary Figure. 4. **Sequence analysis of primary-site mutations in lines 18-f1 and 45-f1.**

(a) A spontaneous 27-bp deletion and 3 nucleotide substitutions within the *tTA* gene in line 18-f1 and **(b)** a spontaneous 26-bp deletion in the *hid*^{Ala5} gene in line 45-f1. Asterisks (*) indicate nucleotide identities, the absence of asterisks indicate nucleotide substitutions (blue) or deletions (maroon), and hyphens (-; maroon) indicate deleted nucleotides with respect to the non-mutated gene sequences above. For 45-f1, the ATG (green) is the translation initiation codon.

a) line 21-f1

5′GTGACCGCCGCCGGGATCACTCTCGGCATGGACGAGCTGTACAAGTAAAGCGGCCGCGACTC
TAGATCATAATCAGCCATACCACATTTGTAGAGGTTTTACTTGCTTTAAAAAACCTCCCACACC
TCCCCCTGAACCTGAAACATAAAATGAATGCAATTGTTGTTGTTAACTTGTTTATTGCAGCTTA
TAATGGTTACAAATAAAGCAATAGCATCACAAATTTCACAAATAAAGCATTTTTTTCACTGCAT
TCTAGTTGTGGCGATCTTGAAGAAGAATGTGTCTTAAAGCTTATCGATACGCGTACGATCCCCG
GGTACCCCTAGAGGGACAGCCCCCCCCCAAAGCCTTTCGGCAGGTAAGTACTTTTTTCCCTGCA
TTCGCGGNGGGGCCGAGTTAAAAGAGAAAACAATCCGGTCCGGCGCTCCCCACTCATCCCCGA
3′

b) line 44-m1

5′GGATCACTCTCGGCATGGACGAGCTGTACAAGTAAAGCGGCCGCGACTCTAGATCATAATCA
GCCATACCACATTTGTAGAGGTTTTACTTGCTTTAAAAAACCTCCCACACCTCCCCCTGAACCT
GAAAAAGGTACTGCAAGTCCCTGCCGATGCGGCCACGACTGCCGTACCACCACCTGGTTACCCG
CCAGAGCTAACTTTAAGCCAAGAATTATCGATGAAGCTACGCCATCGGCAAGAAACTCCAATCC
TTACCTACAGAAGAGCGTCTCGAACGACCCCCCCATTAACCTAGCTAATAGAGACAATAATTTA
GAAAAGAAAATTGACATTTTAATGGCTTTAATCATACAAGGAAGCAATAACAATAATCTTGACA
TTGATACATCCATCTAAATTTACATTACACTTATTTGTATATACATCTATTTAACTATATATAT
CCGCCCCCAAAGCGCACGTCTGTCCAACTTCATAAATTTCTAATTATCATCACCTTTCTCCACG
CAAAGCTTAAACCTCCGTTGAAAAACCAATCCATTAGATGGATGACATTAAAACGTAAATAAAT
GATCTTCTCACCTCAAGCATCCGGATAAAAAAGGGAAGACGCACTCCAACTCCTGATGAAGCTA
TGTGAAGAAAACTACACCAGGATTCAAAAGTC3′

Supplementary Figure. 5. Sequence analysis of primary-site mutations in lines 18-f1 and 45-f1.
Partial insert sequences (shaded black) in the lethal effector vector (maroon) from lines 21-f1 (a) and 44-m1 (b) using TAIL-PCR with forward primers in the ECFP gene (see Supplementary Fig. 3)**.**

```
Query  3    GGTACTGCAAGTCCCTGCCGATGCGGCCACGACTGCCGTACCACCACCTGGTTACCCGCC  62
            |||||| | | |||| |||||  |||| ||||| ||| | | |||||| |   | |||||
Sbjct  436  GGTACTTCTAATCCCAGCCGAGTCGGCAACGACAGCCCTCCGACCACCAGCAGAGCCGCC  495

Query  63   AGAGCTAACTTTAAGCCAAGAATTATCGATGAAGCTACGCCATCGGCAAGAAACTCCAAT  122
            |||||||| ||||||||||||||||||||||||| |||||||||| ||||||||||||||
Sbjct  496  AGAGCTAAATTTAAGCCAAGAATTATCGATGAAGATACGCCATCGCCAAGAAACTCCAAT  555

Query  123  CCTTACCTACAGAAGAGCGTCTCGAACGAcccccccATTAACCTAGCTAATAGAGACAAT  182
            |||||||||||||||||| ||||| |||||||| ||||||||||||||||||||| | |
Sbjct  556  CCTTACCTACAGAAGAGCTTCTCGGACGACCCCACCATTAACCTAGCTAATAGAGTCGAC  615

Query  183  AATTTAGAAAAGAAAATTGACATTTTAATGGCTTTAATCATACAAGGAAGCAATAACAAT  242
            |||||||||||||||||||||||||||||||| ||||||||||||||||| || || |||
Sbjct  616  AATTTAGAAAAGAAAATTGACATTTTAATGGCGTTAATCATACAAGGAAGGAACAATAAT  675

Query  243  AATCTTGACATTGATACATCCATCTAAATTTACATT  278
            |  | |||||| ||||| || | |||||| ||||||
Sbjct  676  A--C-TGACATGGATACTTCAACCTAAATCTACATT  708
```

Supplementary Figure. 6. Blast alignment of the insert sequence in primary-site line 44-m1. Blastn analysis to the NCBI *D. melanogaster* genome database for the line 44-m1 partial insert sequence (Query; see Supplementary Fig. 5B) showing alignment to the *D. melanogaster* HeT DNA sequence (Sbjct) (GenBank ID: M81595.1). Length: 2,837; Identities 239/276 (87%).

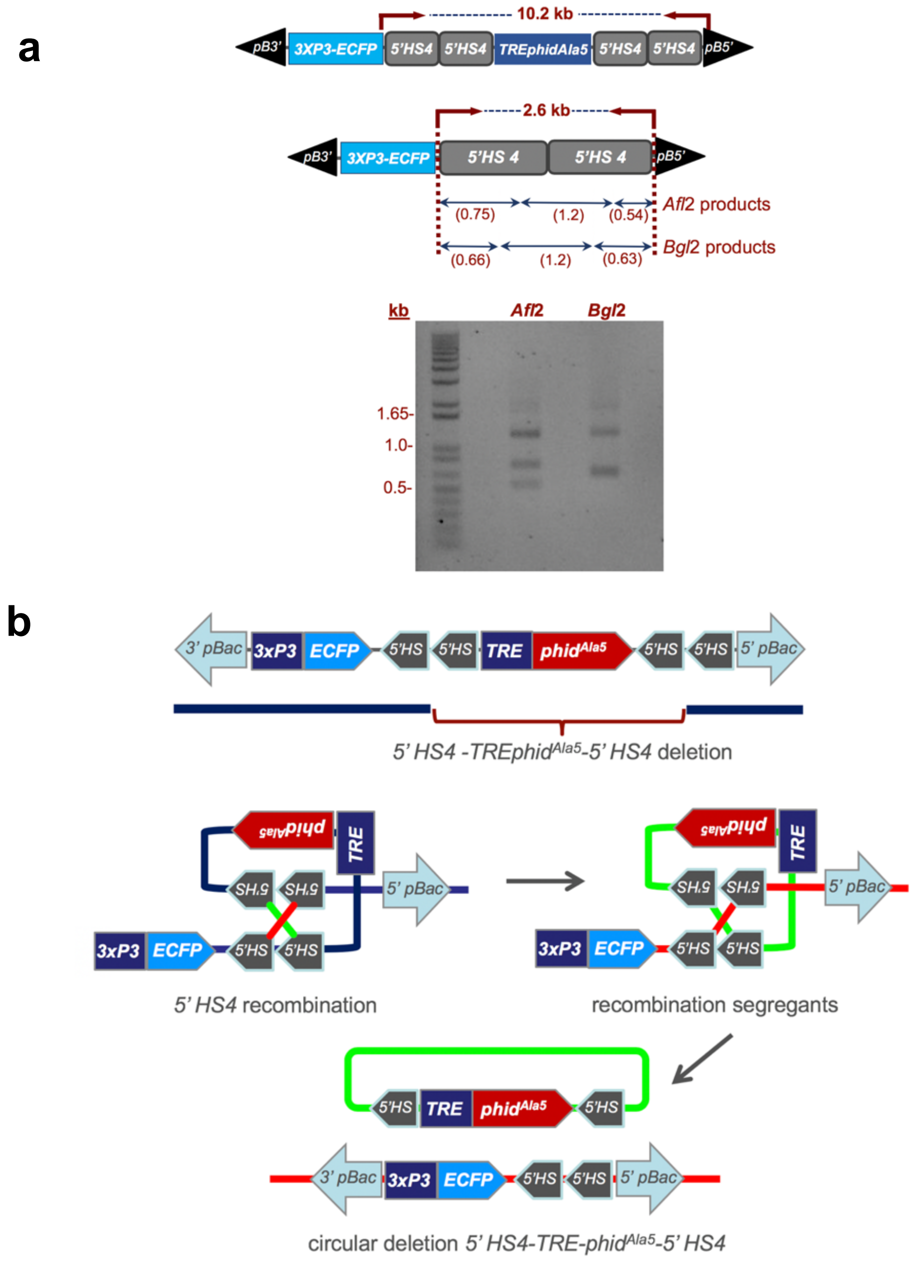

Supplementary Figure. 7. Restriction digest analysis and proposed mechanism for the same internal lethal effector deletion in primary-site lines 9-f1, 62-f1, and 65-m2.
(a) Confirmation of remaining duplicate *5'HS4* insulator sequences in revertant lines 9-f1, 62-f1, and 65-m2 based on *Afl*II and *Bgl*II restriction digest fragments from a 2.6-kb PCR product using primers for *ECFP*-linked *SV40* and *pB*5' (AH1615-AH1566) (see Supplementary Table 2 and Supplementary Fig. 3). The digest pattern observed in all three lines is consistent with a *5'HS4_TREphid*^{Ala5}*_5'HS4* sequence deletion from the lethal effector vector. **(b)** Proposed mechanism for the *5'HS4_TREphid*^{Ala5}*_5'HS4* sequence deletion in the lethal effector vector in lethal revertant lines 9-f1, 62-f1, and 65-m2, by a single *cis*-recombination between paired *5'HS4* insulator sequences within an inverted loop resulting in a circular deletion.
